# Minimally Invasive SPML Surgery for Children with Cerebral Palsy: Program Development

**DOI:** 10.1155/2020/5124952

**Published:** 2020-08-19

**Authors:** Dana L. Wild, Caroline W. Stegink-Jansen, Christine P. Baker, Kelly D. Carmichael, David A. Yngve

**Affiliations:** ^1^Department of Physical Therapy, The University of Texas Medical Branch, Galveston, TX, USA; ^2^Department of Orthopaedic Surgery and Rehabilitation, The University of Texas Medical Branch, Galveston, TX, USA

## Abstract

Improvements in surgical and rehabilitation care are critical to lessen the burden of cerebral palsy (CP), the most common cause of severe physical disability in childhood. The selective percutaneous myofascial lengthening (SPML) surgical procedure is a minimally invasive method designed to improve ambulation by lengthening contracted musculoskeletal tissues. Information on surgical procedures, efficacy, and safety of SPML for children with CP is lacking. Phase 1 of our research is a “proof-of-principle” study for multisite SPML to improve functional mobility of children with CP, and Phase 2 assesses safety, reoperation rates, and efficacy over time in subsequent patient series. Phase 1 was a repeated measurement case series study of 17 children (mean age 7.6 years). One physical therapist, blinded to the surgeon's measurements, measured bilateral knee and ankle motion before and after SPML procedures, using video recordings of a standardized gait path. Functional Mobility Scale (FMS) 5, 50, and 500 outcomes were taken pre- and postoperatively and via telephone follow-up. In Phase 2, multisite SPLM surgeries were implemented in larger successive cohorts from 2006 to 2017. Complications, reoperation rates, and efficacy were retrospectively analyzed. Phase 1 results showed improvement in the children's knee and ankle motion while ambulating and improved FMS 5, 50, and 500 outcomes postoperatively (mean, 6.3 months). At second follow-up (mean 33.3 months), FMS 500 scores continued improvement, while FMS 5 and FMS 50 scores maintained. During Phase 2, the complication rate was 2.4%, and reoperation rates (including reoperations due to maturation) were between 8% and 13%. Improvements to correct ankle equinus were recorded in 498 cases. In conclusion, in a specialized center, single-event, multilevel SPML surgeries of children with CP safely improved ambulatory knee and ankle angle motion and daily mobility outcomes. Future educational studies of training needs for surgeons new to the approach are needed.

## 1. Introduction

Cerebral palsy (CP) is the most common cause of severe physical disability in childhood, with the average prevalence in the United States reported to be 3.6 per 1,000 children [1]. The associated lifetime healthcare cost for these children poses a tremendous burden for caregivers and society [2]. Improvements in surgical and rehabilitation care are critical to improving functional outcomes and quality of life for these patients.

Ambulation is of great importance in fulfilling life roles and optimizing quality of life [3]. Children with CP have difficulties with day-to-day mobility, especially, because many suffer from difficulty walking in home, school, and social settings [4–8]. The effects of neurological and musculoskeletal impairment on walking are usually a key concern of parents. Surgical correction of soft tissue and/or bony structures can optimize the walking ability of many patients with CP. Other goals of surgery in CP are to decrease pain by reducing the effects of spasticity, improve joint alignment, prevent or delay arthritis or progressive deformity, facilitate or decrease orthotic (brace) use, improve self-care and self-esteem, facilitate care, and improve activities of daily living [9].

Surgical interventions create long-lasting changes to the musculoskeletal system, with the intent of enhancing chances for long-term improvements. They do, however, carry risks, including infection or loss of existing function due to immobilization required for healing [10] and pose a burden on the child and caregivers. Consequently, there is a need for minimally invasive surgical procedures that cause minimal scarring and tissue trauma to achieve these goals. This concept has not been completely accepted for children with CP, as there are surgeons who prefer open techniques.

The selective percutaneous myofascial lengthening (SPML) surgical procedure is a minimally invasive method to lengthen contracted musculoskeletal tissues to improve bilateral interactive walking, subsequent functional mobility, and quality of life in children with CP. Developed by RM Nuzzo, SPML differs from other orthopedic surgeries in its aims and procedures [11–14]. Most surgical approaches plan the procedures as a sort of blueprint, with shape and depth, even jigs and patterns used as templates. With SPML, surgeons are not only trying to alter the shape of the musculotendinous unit but also its responsiveness to stretch [14]. The primary goal is to decrease tension, allowing increased ease of motion. Tight musculotendinous units can trigger spasticity. With less tightness, there can be less triggering of spasticity and dystonia.

Advantages of SPML include minimizing the burden of surgery on the child in 2 ways: (1) it makes use of very small incisions not requiring suturing and (2) it is performed with continuous in situ examination by the surgeon, only releasing the minimal amount of myofascial tissue to accomplish the desired lengthening without full disruption of the involved musculotendinous unit.

Although percutaneous surgery in general has a long history going back over 150 years, specific considerations and clinical reasoning algorithms are required when using this technique in children with CP. Specifically, a study of bilateral limb motion occurring during gait is needed to understand the utilization of the acquired length gains during walking [12, 14].

**Figure 1:**
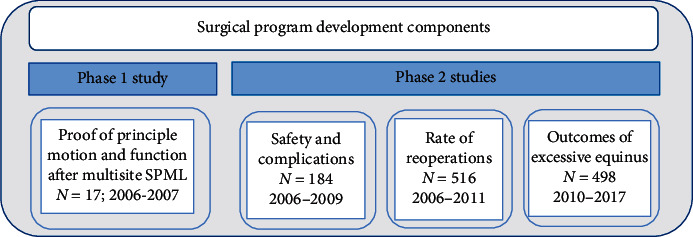
Surgical program development components.

This study follows a progressive, multistep approach for the development of motor interventions [15]. In Phase 1, a “proof-of-principle” analysis is conducted to gain detailed insight into whether and how SPML surgery produces joint-motion changes during walking as intended and to analyze the functional mobility changes in home, school, and social environments over time. The surgical procedures are described. Phase 2 studies looked at complications and reoperation rates and assessed efficacy as cases accumulated from 2006 through 2017.  Figure 1 shows the timeline of the surgical program development.

## 2. Phase 1: “Proof-of-Principle” Study

### 2.1. Patients and Methods

The first step in the program development was to test the feasibility of the minimally invasive surgical technique to lengthen musculotendinous units targeted because they were shortened and showed reflexive spasticity upon stretch, resulting in difficulties with ambulation. Children were enrolled in a study that was part of dissertation research conducted from 2006 to 2007 [16]. The study was approved by the Institutional Review Board at the University of Texas Medical Branch IRB #07-166, Cerebral Palsy Surgery. Parents gave permission for their children to be videotaped. Children with CP were eligible to be included in the study if they had SPML surgery. Other inclusion criteria were being ambulatory (having a Gross Motor Function Classification System [GMFCS] level lower than V) [4–6], availability to attend pre- and postoperative gait video recordings, and completion of the Functional Mobility Scale (FMS) by the caregivers [6]. Subjects undergoing conventional incision open surgery through the duration of the study were excluded. Eligibility for the procedures was established by the surgeons.

Initially, 27 ambulatory children with CP aged 3 to 18 years (mean, 8.4 years) qualified for the study. Children were excluded when only having unilateral releases (*n* = 2), were missing postoperative data (*n* = 5), or poor-quality video recordings obscured by assistive devices (*n* = 2), or biased when the subject was supported by a caregiver during the gait recording (*n* = 1). As a result, 17 subjects were included in the mobility data analysis, with a mean age of 7.6 years (SD 3.3).  Table 1 shows the GMFCS classification, age at the time of surgery, time to first postoperative follow-up of on-site physical therapist measurements, and time to second phone follow-up on a case-by-case basis. All children were followed up through their usual postoperative surgical protocol.

Under general anesthesia, 14 of 17 children had 6 areas lengthened involving 3 levels: ankle, (gastrocnemius), knee (hamstrings), and hip (hip adductor). Three children had 2 levels lengthened bilaterally, at the hip and knee (4 sites). All hip adductor lengthening procedures were accompanied by obturator nerve blocks. No osteotomies, tendon transfers, or larger incisions were performed.

### 2.2. Design

A repeated measurement case series design was used to describe pre- and postoperative range of motion of right and left knees and ankles during ambulation using a standardized gait path. Caregiver-reported functional mobility measures were taken pre- and postoperatively and via telephone at follow-up. Follow-up 1 took place 5 to 13 months postoperatively (mean, 6.3 months; median, 5 months). Follow-up 2 via telephone took place 27 to 38 months postoperatively (mean, 33.3 months; median, 33 months). Motion measurements were taken by 1 physical therapist, independent of the operating surgeon and blinded to the surgeons' examination measurements, as part of dissertation research conducted from 2006 to 2007 [16]. All gait data were collected in the same space using a standardized protocol under specifically controlled clinical environment conditions of walkway and positioning and location of the camera to minimize introduced error. The SPML surgeries were performed by 2 surgeons, both trained in SPML techniques [13, 14].

### 2.3. Surgeon's Procedures and Perioperative Protocols

#### 2.3.1. Preoperative Procedures

Preoperatively, the surgeon determined the position at which reflexive spasticity was triggered by measuring the joint angle of the hips, knees, and ankles at the initial grab when a fast, gentle stretch was applied.

#### 2.3.2. Surgical Intervention Procedures

The difference between the clinical exam and the exam under anesthesia informs the surgeon on the amount of lengthening needed. The decision to correct fixed contractures that are present both awake and asleep is straightforward, but the decision to correct dynamic contractures, with hyper reflexivity, spasticity, and dystonia resulting from the neurological disorder, is not. Dynamic contractures can be severe and problematic, and, in that respect, worthy of treatment. They can be present when awake but reduced when asleep or under anesthesia. Thus, surgically reducing dynamic contractures carries a greater risk of overcorrection compared with surgically reducing fixed contractures. These factors need to be acknowledged and carefully weighed to avoid overcorrection that could jeopardize the positive outcome of the surgery.

The following safeguards were applied to ensure the accuracy and safety of the procedures. First, anatomical determination of the location of the individual muscle-tendon units was confirmed by palpation and listed by name. Second, anatomical awareness of adjacent local neurovascular structures was kept in mind.

Standardized intraoperative surgical procedures included (1) supine positioning of the child, (2) determination of the areas for the incisions, (3) determination of the palpably tight structures by location and name, (4) performance of the release surgery, and (5) continually assessing the tension over the range of motion as the release was being performed to prevent over-lengthening.

The major emphasis of the surgical procedures is not targeted to increasing range of motion, but instead to reducing tension in the musculotendinous units to allow for increased ease of motion, without over-lengthening. The goal of SPML procedures is not a *premeditated* exact shape, direction, size, or any exact geometric feature. The goal in the clinic is a process of evocative discovery, standardized tests, and provocative maneuvers to test the presence and impact of excess reactivity. This involves not only checking reflexes but observing how reactivity such as spasticity in one area triggers movements or postures that compromise function in other areas. If the surgeon can reduce this chain of excess reactivity, there can be a reduction in the source of function loss. Since excess reactivity in one location can spread to another location, the neurologic spread of dysfunction from maladaptive abnormal reflexes can be reduced by properly targeted SPML procedures [17].

Gastrocnemius myofascial lengthening is typically performed in the low calf at the distal level of the muscle belly. Hamstring surgery includes 1 to 4 of the following as indicated through clinical examination: semitendinosus tenotomy, myofascial lengthening of gracilis, semimembranosus, and biceps femoris. The level of surgery is in the distal third of the thigh, usually within several centimeters of the popliteal crease. Hip adductor tenotomy is performed close to the groin crease. In all cases, number 6500 Beaver blades (Becton Dickinson & Company, Waltham, Massachusetts) were used. It is very helpful to use a blade with a sharp, pointed tip. This helps the surgeon sense tight fascial structures through the fingers as they grasp the knife handle, through haptic feedback. One to 3 incisions 1 to 3 mm long were made at each area through which the releases were performed. When performing release surgery, the guiding principle is, “When in doubt, do less.”

Obturator nerve blocks are used if the child is demonstrating a scissoring gait pattern, the child is reactive to adductor stretch, or if hip dysplasia is evident on anteroposterior pelvis radiographs. The obturator nerve block is typically performed using 3 mL of 50% ethanol, in conjunction with a nerve stimulation needle. This concentration of ethanol is a relatively gentle agent compared with phenol. The application of ethanol perineural focal dysmyelination is a logical part of this method as it targets dampening of reflexive abnormal movements. The obturator nerve is primarily a motor nerve. Nerve blocks to mixed motor and sensory nerves have the possibility of chronic pain and are thus contraindicated. Phenol was not used.

#### 2.3.3. Postoperative Procedures

Postoperative care included the application of short leg casts with the ankles at 0 to 7 degrees of dorsiflexion (0 to −7 degrees of equinus) for 4 weeks to maintain the gastrocnemius lengthening. The use of loosely fitted knee immobilizers at night to prevent sleeping in the fetal position was recommended for 1 month to maintain lengthening of the hamstrings. No immobilization was recommended to maintain hip adductor lengthening. Typically, children are allowed to start ambulation the day of surgery and strongly encouraged to start by the third day. For pain and spasticity control, over-the-counter oral acetaminophen and oral diazepam 2 mg are prescribed to be used as needed. Children are allowed to return to their usual occupational and/or physical therapy immediately following the procedures, with no restrictions.

### 2.4. Outcome Measurements

#### 2.4.1. Video Gait Analysis

Each subject had a preoperative and a postoperative video recording made in the outpatient clinic of a children's hospital, in a standardized space of 2 crossing hallways. A Sony Digital Handycam DCR-TRV 130 NTSC560x camera (Sony Corporation of America, New York, NY) fixed on a tripod was positioned 4.2 m from the subject. The camcorder viewing area was 1.2 m, with a 1.2 m walk-in and a 1.2 m walk-out area. Video recording was taken with the child walking left to right, and another was taken walking right to left to ensure that each individual lower extremity could be well visualized. All measurements were taken from the side.

**Figure 2:**
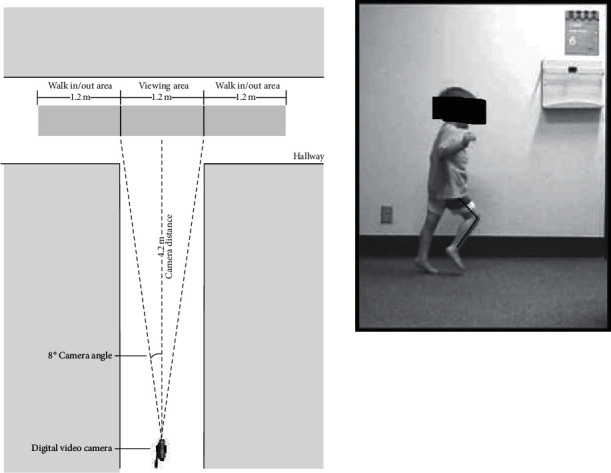
Camera setup and example of drawing the range-of-motion lines.

The video recordings were loaded into a BioGait software program (Seaside Software, Berlin, MD, USA), currently available through SportsCAD [18]. This program permits frame-by-frame analysis to ensure that the measurements are taken at the appropriate time during the gait cycle. The knee and ankle angles were measured by using the mouse to draw lines representing the thigh and the lower leg, or the lower leg and the foot. The software calculated the angles between the lines.  Figure 2 shows an example of a knee measurement with the angle marked by lines. Accuracy and inter- and intra-rater reliability were tested and approved [15] prior to the data collection and matched outcomes in other reports [17].

A total of 6 parameters were measured and recorded. For the knee, (1) maximum flexion in swing, (2) minimum flexion (=maximum extension) at terminal swing, and (3) minimal flexion (=maximum extension) in stance; for the ankle, (4) maximum equinus at toe-off, (5) minimum equinus (=maximum dorsiflexion) in swing, and (6) minimum equinus (=maximum dorsiflexion) in midstance. Two additional parameters were derived from the measured angles: knee excursion (knee flexion in swing minus knee flexion [lack of extension] in stance) and ankle excursion (equinus at toe-off minus equinus in stance) [17].

#### 2.4.2. Functional Mobility Scale (FMS)

The FMS was used to evaluate the children's walking ability, taking into consideration the use of crutches or walkers at home (5 m), at school (50 m), and at a large store or shopping mall (500 m) [6]. The FMS is a reliable and valid tool for demonstrating changes in functional mobility after orthopedic surgery [6–8]. FMS scores were obtained before surgery and at first postoperative follow-up in the clinic (mean, 6.2 months; SD, 3 months). A second follow-up was conducted with the caregiver or parent via telephone interview for 15 children and during a clinic visit with the surgeon for 2 children (mean, 33.3 months; SD 3.4 months; median, 33 months; range, 27–38 months). The FMS has been validated for use by telephone interview [19] and has been validated for true performance when reported by parents [8].

#### 2.4.3. Data Analysis

IBM SPSS Statistics 25 was used to analyze the data. Descriptive analyses were conducted for all outcome measures.

#### 2.4.4. Knee and Ankle Joint Motion Measurements during Ambulation

Descriptive statistics were calculated for the knee (flexion in swing, terminal swing, and in stance) and ankle (equinus at toe-off, in swing, at terminal swing, and in stance). Two-factor repeated-measures ANOVA analyses were performed to analyze 6 knee joint range-of-motion (ROM) parameters and 2 derived parameters: 1 factor for pre and post comparisons and 1 factor for right and left comparisons. After screening of the data, nonparametric Wilcoxon signed-ranks tests were used to test the differences between pre- and post-test ankle ROM measurements for right and left legs separately. Given the explorative nature of the study, no Bonferroni adjustments were applied [20].

#### 2.4.5. Functional Mobility Scale (FMS 5, FMS 50, and FMS 500)

To test progression over time for the FMS 5, FMS 50, and FMS 500, the Friedman test was applied for differences between 3 time points: preoperative baseline, follow-up 1 in clinic, and follow-up 2 via telephone. Follow-up analyses between paired time points were performed using the Wilcoxon signed-ranks test [20].

## 3. Phase 1: “Proof of Principle” Results

### 3.1. Knee and Ankle Range of Motion Measured during Ambulation

#### 3.1.1. Knee Angle Results

Table 2 lists the descriptive statistics of knee ROM measured during ambulation of the right and left legs, preoperative and postoperatively.  Table 3 shows the inferential results for the knee, including effect sizes.

**Figure 3:**
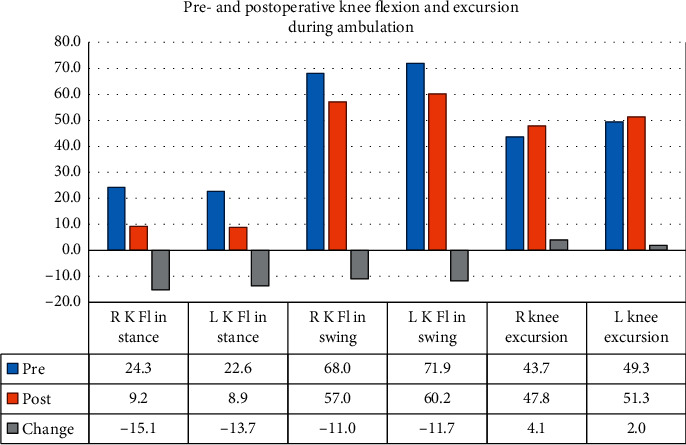
Knee flexion during ambulation shows an improvement in knee flexion in stance and a decrease in knee flexion in mid swing (*N* = 17). Knee excursion during ambulation (between stance and mid swing) remained stable but shifted to a more desirable range in the gait cycle arc.

Knee flexion significantly decreased for all measurements (knee flexion during swing, terminal swing, and in stance) (*P* < 0.05). Effect sizes ranged between 0.4 and 0.75. Differences between right and left legs were not significant (*P* > 0.05). The interaction effect was also found to be statistically not significant. Decreases in flexion (extension lag) in terminal swing and stance signify improvement; a decrease in knee flexion in swing signifies a worsening of knee flexion during swing.  Figure 3 shows the pre- and postoperative mean knee flexion values and the associated mean change between pre- and postoperative knee flexion.

#### 3.1.2. Ankle Angle Results: Data Screening

In screening of the data, 5 subjects preoperatively walked with a negative equinus gait (crouch gait) on the right and 3 of those 5 also had a negative equinus gait on the left. Intraoperative examination showed gastrocnemius shortness in these five children, which necessitated lengthening procedures. Consequently, for subjects with a baseline positive equinus, decreases of equinus in stance, swing, and terminal swing indicate an improvement; whereas for subjects with a negative equinus, a further decrease of equinus would be considered a worsening. Therefore, the analyses for ankle ROM were performed for baseline right positive and baseline right negative equinus subjects separately.

**Figure 4:**
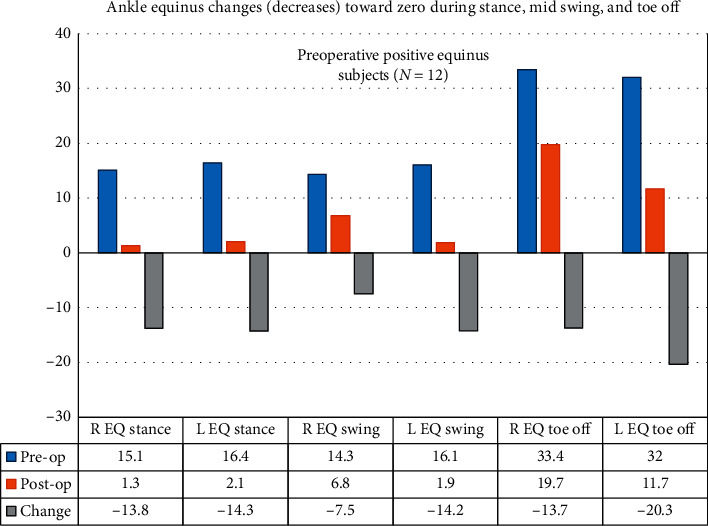
Twelve subjects had preoperative positive equinus ankles. Ankle equinus (equinus in stance, mid swing, and toe-off) for right positive equinus subjects shows a decrease (improvement) in equinus toward zero in all parameters.

**Figure 5:**
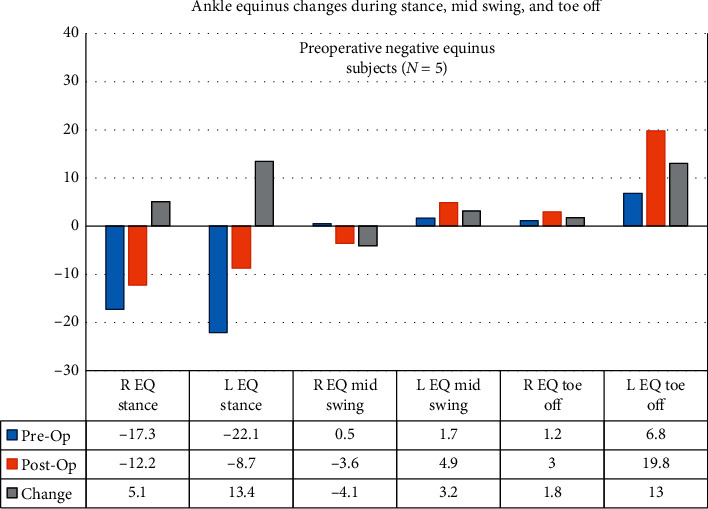
Five subjects had preoperative negative ankle equinus during ambulation. Equinus in stance increased toward zero in stance, decreasing crouch gait pattern. Responses varied for right and left legs for mid swing. Equinus increased during toe-off.

Table 4 lists the descriptive statistics of ankle ROM for positive and negative equinus subjects measured during ambulation of the right and left legs, preoperative and postoperatively.  Table 5 shows the inferential results for the ankle, including effect sizes. For the 12 right equinus-positive subjects, equinus at toe-off, equinus at swing, equinus at terminal swing, and equinus in stance decreased (improved) significantly (*P* < 0.05) for the right and left legs, with the exception of right ankle equinus during swing (*P*=0.126). Effect sizes ranged between 0.38 and 0.65 for the right leg and between 0.62 and 0.78 for the left leg. For the 5 right equinus-negative subjects, none of the ankle measurements showed significant changes between pre- and postoperative measurements, with the exception of right ankle equinus (*P*=0.042), with an effect size of 0.67. Figures 4 and 5 summarize the ankle measurement changes for the preoperative equinus-positive and equinus-negative subjects, respectively.

#### 3.1.3. Derived Measures of Excursion Results

Table 6 presents mean and standard deviations of calculated knee and ankle excursions for baseline and post-test measurements. The excursion baseline and post-test comparisons were not significant, with the exception of the 12 right equinus-positive subjects postoperative left ankle excursion, which was less than preoperative left ankle excursion (*P*=0.03). None of the excursions for the right equinus-negative subjects changed significantly between pre- and postoperative measurements.

#### 3.1.4. Functional Mobility Scale (FMS 5, FMS 50, and FMS 500)

Table 1 shows the FMS scores per GMFCS level. Subjects with GMFCS II showed preoperative FMS between 3 and 5, with GMFCS level III showing scores between 1 and 4, and those with GMFCS level IV exhibiting functional scores between 1 and 2.  Table 7 shows the median and range of the FMS for all 3 time points for each GMFCS level and the inferential results between the 3 time points. The FMS 5, 50, and 500 scores improved over time: from a median of 2 to 5 for the FMS 5, a median of 2 to 3 for the FMS 50, and a median of 1 to 3 for the FMS 500. The Friedman test showed significant differences between the 3 time points. Follow-up by Wilcoxon signed-rank tests showed that the FMS scores improved between preoperative and postoperative measurement and remained stable between postoperative and follow-up measurements for the FMS 5 and FMS 50. Subjects continued to improve significantly on the FMS 500 between the first and second follow-up. At follow-up, all subjects showed significant improvement on the FMS compared with all preoperative FMS scores.

### 3.2. Phase 1 Complications

There were no wound infections, reports of nerve damage, sensory loss, weakness, dysasthesia, or reports of blood vessel damage. One patient had a postoperative transient fever, possibly due to an ear infection. Postoperative pain was easily controlled with analgesics. One patient underwent serial casting of the knees to treat residual flexion contractures. Two patients had tight casts, one was relieved by removing the proximal 5 cm of the cast and the other by splitting the cast anteriorly at the midline.

### 3.3. Transition Decision to Move from Phase 1 to Phase 2

The “proof-of-principle study” demonstrated knee and ankle mobility significantly improved, as well as functional mobility in daily life indicated by the FMS scores. Long-term gains were maintained or continued to improve for more demanding tasks. Safety concerns were also met. Therefore, Phase 2 could be started with implementations of the SPML lengthening approach to cohorts.

## 4. Phase 2 Studies: Implementation of the Surgical Program (2006–2017)

Three subsequent SPML research studies were performed to assess complication, reoperation rates, and efficacy in larger cohorts of subjects, as the surgical program described above was implemented [21–24].  Figure 1 shows the time lines of the studies and the accumulation of cases.

### 4.1. Safety and Postoperative Complications (Subjects from 2006 to 2009) [22]

A longitudinal study was conducted to assess the prevalence of postoperative complications of the SPML procedure when applied to children with CP using a retrospective review of all charts and/or electronic medical records of patients aged 2 to 18 years with CP who had SPML surgery between 2006 and 2009. A total of 184 children (101 boys and 83 girls) with a mean age at time of surgery of 8.9 years underwent a total of 1102 individual SPML procedures. All patients were 1 year or more postoperative at the time of data collection. The number and type of complications were recorded. The results showed 27 reported complications (2.4%), which included 1 case with fever, 2 hematomas, 8 paresthesias, 4 tight casts, 11 flexion contractures, and 1 ruptured muscle. The ruptured muscle was the gracilis and presented with pain and swelling. These issues were resolved when reassessed after 2 months. There was no loss or decrease of function. None of these complications required hospital admission for treatment or resulted in chronic pain.

### 4.2. Rate of Reoperations (Subjects from 2006 to 2011) [23]

The rate of reoperation at 1- to 6-year follow-up was determined via retrospective chart analysis for 516 children with CP treated with SPML between 2006 and 2011. Reoperation rates were analyzed by age and joint region (hip, knee, or ankle). The 4 age groups, based on important child development stages, were 2 to 5 years of age (123 patients), 6 to 9 years (156 patients), 10 to 13 years (128 patients), and over 14 years (109 patients). The results show that reoperation rates were the highest (13%) in patients in the youngest group, and 12%, 9%, and 8% for those in the second, third, and fourth groups, respectively.

### 4.3. Minimally Invasive SPML Surgery in a Larger Cohort to Improve Ankle Equinus

After the complication and reoperation rates were deemed acceptable, the procedures were implemented to treat a larger number of patients with SPML for equinus correction of the ankle (subjects from 2010 to 2017) [24]. Percutaneous release for equinus correction was performed in 498 consecutive patients, age ranging from 1 to 42 years (median, 6 years) from 2010 to 2017. The GMFCS levels included level I: 52 patients, level II: 173, level III: 65, level IV: 31, and level V: 177 patients. This study showed the interactive nature of the procedures. The surgical technique principle was to start the release at the gastrocnemius level and then work distally if needed. If equinus deformity persisted after gastrocnemius release, additional release was done at the soleus level; if deformity continued to persist, release was performed at the Achilles level. No posterior capsulotomies were performed during this consecutive series. As a result, the gastrocnemius fascia was released in 673 extremities (77%), the gastrocnemius and soleus fascia in 86 (10%), and the Achilles tendon in 112 (13%) extremities. Successful correction was defined as correction of ankle equinus to 0 degrees with the knee flexed in the operating room, which would allow for cast application with the ankle in a neutral position. Results showed that intraoperative correction to neutral was achieved in 829 of 871 ankles (95%). The success rate varied by initial preoperative severity of the ankle equinus deformity from 99% (10 to 19 degrees equinus deformity) to 50% (70 to 80 degrees of equinus deformity). Patients were 71% less likely to have a correction to neutral if the preoperative ankle equinus with the knee flexed was ≥30 degrees.

## 5. Discussion

This series of investigations offers evidence that multilevel, simultaneously applied SPML techniques can safely and successfully be used as stand-alone procedures in appropriate clinical scenarios, and the use of open surgery may not be mandated in all circumstances. The approach follows a model of stepwise progressing of clinical studies recommended in the literature [15]. Our results support other reports of percutaneously performed surgeries to lengthen Achilles tendons [25], medial hamstring tenotomies [26], soft-tissue releases of the hip adductor with or without anterior obturator neurectomy [27], and adductor releases [28].

The Phase 1, proof-of-principle case series study showed surgical lengthening translated into desired increases of the knee and ankle motion during ambulation. Insight was also gained into how SPML releases can change affected gait parameters. Knee extension improved during the knee extension phases of ambulation, but knee flexion decreased in the knee flexion phase of ambulation after the procedures. Interestingly, this resulted in a total knee flexion excursion of the knee, which was similar in quantity, but was shifted to a more beneficial arc of motion. Similar findings were found for the ankle motions. Functional daily mobility measured by the standardized FMS showed significant improvements that were maintained over time once they were achieved and continued to improve for the more demanding tasks of larger distance ambulation in the community.

Phase 2 implementation studies of the surgical program demonstrated safety and efficacy in larger cohorts of subjects. The safety study showed a low complication rate of 2.4%, none of which required reoperation [21]. The reoperation rate study demonstrated a reoperation rate of 8% to 13% with 1- to 6-year follow-up [22]. The equinus study showed the good results of a 7-year experience in correcting equinus. A desirable outcome was that in 87% of cases, surgery was limited to the gastrocnemius and soleus. No Achilles tendon surgery was performed in this group [23].

Studies addressing surgical interventions to lengthen musculotendinous units are increasing, with a variety of surgical techniques being developed. The SPML technique, initiated by Nuzzo [13, 14] and used in this series of studies, is characterized by being a minimally invasive and interactive approach, with a focus on (1) lengthening at the myofascial location, rather than the tendinous region, and (2) intraoperative assessment of length gains to minimally disturb involved tissues. Since the technique still is considered innovative, current reported series are small, may fully or partially use the SPML technique, and outcome measures may vary. A retrospective study of 41 children by Davids et al. reported on an intraoperative, interactive approach of slowly lengthening the medial hamstrings; however, this technique was open, rather than minimally invasive [10]. As in other developing innovations, further studies are indeed needed comparing pre-, intra-, and postoperative approaches in a systematic manner [15].

No consensus exists about the ideal outcome measures to quantify outcomes. One outcome study of SPML focused on improved gross motor function after the intervention in a group of 58 children, but the study did not include a study of joint motion during gait analysis [29]. Most recently, a report including 10 children treated on multiple sites confirmed improvements in gross motor function, muscle strength, and step length. Joint mobility data were collected but not reported [11]. No studies that assess joint excursion after lengthening surgeries were found. This derived measure provided insight into the changed joint motion during ambulation in the proof-of-principle study.

No consensus exists to analyze right and left legs separately or combine those as one sample of legs rather than subjects. Some authors join right and left measurements, which has the advantage of increasing the participation numbers to enhance power for statistical analyses. A multisite study of open surgical lengthening releases by 14 surgeons from 31 patients (39 sides) with CP with unilateral and bilateral impairments combined right and left leg values [33]. Significant improvements in knee extension were found, similar to our outcomes, but knee flexion in swing was maintained in contrast to the decreased knee flexion found in our study. Ankle dorsiflexion improvement, a decrease in positive equinus, was similar to that in our study. Even though we did not find differences between right and left leg values, we did not combine right and left measurements and analyzed joint motion for the right and left separately. Further studies are needed to determine whether these values can be combined to provide a larger sample size or cannot be combined due to their compensatory interdependency [12].

Our findings suggest caution in the interpretation of the ankle equinus position. With a neutral ankle position as a milestone, equinus measurement will decrease as patients with a positive equinus improve but may increase, therefore becoming less negative, for patients with a crouch gait. This phenomenon does not occur in the knee, as recovery of knee motion occurs in the same direction for both gait patterns. Other studies also categorized the different gait patterns and defined crouch by the appearance of knee motion [30–32]. Further study is needed to determine the best choice for analyzing joint angle data in the context of different gait patterns.

No information was found regarding postoperative care protocols for multisite simultaneous open releases, clinical practice guidelines for immobilization, time to resume ambulation, weight bearing status, or participation in life roles and activities. In this study, all surgeries were done on an outpatient basis. Orthopedic postsurgical pain is often a concern for parents [33–35], and the use of oral pain medications was sufficient. Short leg casts with the ankles at 0 to 7 degrees of dorsiflexion were used for 4 weeks after surgery to maintain length after gastrocnemius lengthening. The use of loosely fitted knee immobilizers was recommended for 1 month at night only, simply to prevent sleeping in the fetal position, after lengthening of the hamstrings. No immobilization was recommended following hip adductor lengthening. Children were allowed to begin ambulation the day of surgery and encouraged to start ambulation by the third day. Some children voluntarily stood and took steps on the first day; all voluntarily did so by 1 week, thereby utilizing length gains of knee motion early on. Children were allowed to return to their therapy immediately following the procedures with no restrictions.

Our study had limitations. The burden of this minimally invasive approach on the patient and family was not tested in a randomized controlled setting against lengthening procedures that require large skin incisions and more extensive releases. Operative scarring has been reported as the number one concern of parents after conventional, multilevel surgery [35], and residual scarring from the SPML approach was found to be minimal by the parents.

Maturation may have played a role in these longitudinal mobility studies of children in Phase 1 and Phase 2 studies. Rosenbaum et al. postulated that 90% of the gross motor function in children with CP is gained by 5 years of age [36]. Maturation of joint ROM of the knee and ankle during gait has been reported for children as young as 5 years of age [37], with children of 5 years already exhibiting ankle and knee flexion and extension similar to those of an adult comparison sample, even though other parameters, such as velocity and displacement, were dissimilar. Two participant children were 3.8 and 4.2 years old, respectively, at the time of baseline measurements. The first child had FMS scores of 2 for the FMS 5 and 50 and a score of 1 (wheelchair) for the FMS 500 at baseline, maintained the same scores for the FMS 5 and 50 but increased to a 2 for the FMS 500 score at first follow-up, and reached a 6 in all 3 categories at second follow-up. The second child began as a wheelchair user for all distances and by first follow-up had improved to a 5 for the FMS 5, 2 for the FMS 50, yet remained a 1 (still a wheelchair user) in the FMS 500. This child scored 6 in all FMS categories by second follow-up. Further studies are needed to examine the interaction between early intervention and hastening of gains in functional mobility and joint-angle measurement during ambulation [38].

A choice was made to include a set of measures, rather than all measures one could take. The mobility measures were active joint angle measures during ambulation, so the postoperative impact of passive joint motion [17] could not be determined. We did not take measures of spasticity [36, 39]. We cannot assume that the changes in functional joint mobility caused the changes in the FMS outcomes and observations by the parents.

Future research is needed to further develop this minimally invasive technique. Multicenter outcome studies, collaborative decision-making for optimal outcome measures, and basic science studies are needed, as are studies on the recovery of muscle function in the growing child. Particularly worthwhile would be exploring whether, through the lengthening procedures, length-tension relationships may change toward a more efficient use of the musculature [40, 41]; whether the intervention changes the path of GMFCS, altering a long-term prediction of function; and if the ankle- and knee-motion improvements were clinically strong enough to change gait from a pattern of haltingly performed steps into continuous walking [14].

Caution is advised in the continued development of the use of SPML procedures. The goal is to improve outcomes for children with CP with carefully planned and executed procedures. The surgeons in this series of studies were familiar with the anatomy through past experience in performing the procedures open. They were both thoroughly trained in the percutaneous procedures. Future educational studies need to develop competency criteria for the performance of these minimally invasive procedures. Our findings can only be generalized to surgeons with adequate proficiency.

## 6. Conclusions

Our findings hold promise for children with CP and their parents, showing that children during ambulation indeed use the muscle-length gains as intended, as demonstrated by the goniometric measurements. In addition, subjects representing a range of levels of functional impairments improved their functional mobility in the environment. Subsequent program implementation studies support the safety of the minimally invasive SPML release of the lower extremities based on the low complication and low reoperation rates. Future studies may determine the place of SPML techniques within the context of conventional methods and, if studies include multicenter collaborations between surgeons skilled in the techniques, may investigate the optimal age for these interventions [38] to enhance the development of children with CP and provide hope and encouragement to their caregivers.

## Figures and Tables

**Table 1:** Subject demographics. Preoperative Functional Mobility Scale (FMS)^*∗*^ ratings at 5, 50, and 500 meters and 2 follow-up times, listed by Gross Motor Function Classification System (GMFCS)^*∗∗*^ level.

GMFCS level	Preoperative					Postoperative	
	Gender (M/F)	Age (years)	FMS 5 (home)	FMS 50 (school)	FMS 500 (shopping center)	Follow-up 1 (months)	Follow-up 2 (months)
Level II	F	5	4	4	4	10	29
	M	7	5	5	5	5	33
	M	6	3	3	3	4	38
	F	7	5	5	4	3	36
	M	17	5	5	5	4	37

Level III	M	6	2	2	1	4	29
	F	4	2	2	1	8	31
	F	13	2	2	1	4	30
	F	7	2	2	1	13	35
	M	9	4	4	1	4	32
	F	10	2	2	1	5	37

Level IV	M	4	1	1	1	8	38
	M	9	1	1	1	12	33
	F	6	2	2	1	5	34
	M	8	1	1	1	6	27
	M	7	2	1	1	5	32
	M	6	1	1	1	5	35

^*∗*^Functional Mobility Scale (FMS 5, 50, 500) : 1 = wheelchair; 2 = walker/frame; 3 = crutches without help; 4 = canes without help; 5 = independent level surfaces; and 6 = independent all surfaces. ^*∗∗*^Gross Motor Function Classification System: 1 = independent; 2 = stair rail; 3 = in/out with assistive device; 4 = walker; and 5 = wheelchair. (median 3, range 2–4).

**Table 2:** Mean and standard deviations (in parentheses) of knee flexion in swing, terminal swing, and stance.

	Right pre-op, *N* = 17	Right post-op, *N* = 17	Left pre-op, *N* = 17	Left post-op, *N* = 17
Knee flexion, swing	68.0 (14.1)	57.0 (10.6)	71.9 (11.4)	60.2 (12.4)
Knee flexion, terminal swing (extension lag)	40.3 (15.6)	23.6 (11.4)	44.1 (14.2)	25.7 (9.7)
Knee flexion, in stance (extension lag)	24.3 (20.8)	9.2 (11.4)	22.6 (20.0)	8.9 (13.1)

**Table 3:** MANOVA pre- and postoperative comparisons of right and left knee flexion in swing, knee flexion at terminal swing, knee flexion in stance. Significance of difference between preoperative and postoperative values.

	Pre-op to post-op	Effect size pre-op to post-op	Right to left	Interaction
Knee flexion, swing *N* = 17	*P*=0.005 Decrease	0.4	*P*=0.24	*P*=0.84
Knee flexion, terminal swing (extension lag) *N* = 17	*P* < 0.001 Improvement	0.75	*P*=0.23	*P*=0.59
Knee flexion, in stance (extension lag) *N* = 17	*P* < 0.001 Improvement	0.65	*P*=0.64	*P*=0.67

**Table 4:** Mean and standard deviations (in parentheses) of ankle equinus at toe-off, swing, terminal swing, and in stance. Children were designated as positive or negative equinus based on the equinus in stance of the right leg.

Children with pre-op right positive equinus in stance	Right pre-op, *N* = 12	Right post-op, *N* = 12	Left pre-op, *N* = 14	Left post-op, *N* = 14

Ankle equinus, toe-off	33.4 (15.1)	19.7 (13.8)	32.0 (20.1)	11.7 (11.2)
Ankle equinus, in mid swing	14.3 (11.8)	6.8 (7.2)	16.1 (9.3)	1.9 (5.6)
Ankle equinus, terminal swing	21.0 (13.1)	6.3 (7.8)	19.6 (12.1)	6.1 (6.7)
Ankle equinus, in stance (dorsiflexion lag)	15.1 (11.1)	1.3 (5.2)	16.4 (12.4)	2.1 (4.7)

Children with pre-op right negative equinus in stance	Right pre-op, *N* = 5	Right post-op, *N* = 5	Left pre-op, *N* = 3	Left post-op, *N* = 3

Ankle equinus, toe-off	1.2 (11.9)	3.0 (10.6)	6.8 (23.8)	19.8 (21.7)
Ankle equinus, in mid swing	0.5 (15.5)	−3.6 (13.3)	1.7 (15.1)	4.9 (10.8)
Ankle equinus, terminal swing	9.2 (7.7)	1.4 (10.7)	13.7 (8.1)	9.3 (4.7)
Ankle equinus, in stance (dorsiflexion lag)	−17.3 (9.4)	−12.2 (9.3)	−22.1 (2.3)	−8.07 (13.2)

**Table 5:** Pre- and postoperative comparisons of ankle equinus at toe-off, swing, terminal swing, and in stance using Wilcoxon signed-rank tests (*P* values), separated by right and left cases with positive and negative equinus in stance at baseline.

Ankle Results	Right *P* value	Right effect size (Eta^2^)	Left *P* value	Left effect size (Eta^2^)
Right positive equinus subjects (*N* = 12)				
Ankle equinus, toe-off	*P*=0.028 Decrease	0.38	*P*=0.002 Decrease	0.78
Ankle equinus, swing	*P*=0.126 NS	NA	*P*=0.002 Improvement	0.68
Ankle equinus, terminal swing	*P*=0.005 Improvement	0.65	*P*=0.005 Improvement	0.62
Ankle equinus, in stance	*P*=0.002 Improvement	0.6	*P*=0.003 Improvement	0.7

Right negative equinus subjects (*N* = 5)	Right		Left	
Ankle equinus, toe-off	*P*=0.684	NA	*P*=0.500	NA
Ankle equinus, swing	*P*=0.345	NA	*P*=0.500	NA
Ankle equinus, terminal swing	*P*=0.042 Decrease	0.67	*P*=0.68	NA
Ankle equinus, in stance	*P*=0.225	NA	*P*=0.225	NA

**Table 6:** Mean and standard deviations (in parentheses) of knee and ankle joint angle excursion for the right and left knees and ankles. Knee excursion is defined as knee flexion in swing minus knee flexion in stance. Ankle joint angle excursion is separated by right positive and negative equinus-mediating factor in stance at baseline. Ankle excursion is defined as equinus at toe-off minus equinus in stance.

Excursion	*N*	Right pre-op	Right post-op	Left pre-op	Left post-op
Knee excursion	17	43.7 (17.3)	47.8 (15.8)	49.4 (20.0)	51.4 (18.2)
Ankle excursion (*R* positive equinus)	12	18.3 (19.1)	18.4 (13.9)	18.0 (10.7)	9.6 (11.2)^*∗*^ Decrease
Ankle excursion (*R* negative equinus)	5	18.5 (7.6)	15.2 (12.4)	17.9 (21.4)	20.6 (17.3)

^*∗*^The only significant difference: a decrease between pre- and post-test ankle excursion for *L* ankle, *P*=0.03.

**Table 7:** Median and range of preoperative Functional Mobility Scale ^*∗*^(FMS) ratings and at postoperative follow-ups 1 and 2 and results of statistical analyses. The Friedman analyses indicate overall difference between the 3 time points; follow-up tests with the Wilcoxon signed-ranks test indicate differences between pairs of time points.

FMS scores	Pre-op median (range)	Follow-up 1 median (range)	Follow-up 2 median (range)	Friedman test (*N* = 17)	Wilcoxon pre-op to follow-up 1 (*N* = 17)	Wilcoxon follow-up 1 to follow-up 2 (*N* = 17)	Wilcoxon pre-op to follow-up 2 (*N* = 17)
FMS 5 meters (home)	2 (1–5)	4 (2–6)	5 (1–6)	*P*=0.001	*P*=0.004	*P*=0.13	*P*=0.03

FMS 50 meters (school)	2 (1–5)	2 (2–5)	3 (1–6)	*P*=0.003	*P*=0.005	*P*=0.12	*P*=0.05

FMS 500 (shopping center)	1 (1–5)	2 (1–5)	3 (1–6)	*P* < 0.005	*P*=0.002	*P*=0.04	*P*=0.01

^*∗*^Functional Mobility Scale: 1 = wheelchair; 2 = walker/frame; 3 = crutches without help; 4 = canes without help; 5 = independent level surfaces; and 6 = independent all surfaces.

## Data Availability

The data used in this study will be provided upon request.
